# Accurate quantitation of antibiotic penetration through staphylococcal biofilms

**DOI:** 10.1016/j.bioflm.2025.100316

**Published:** 2025-09-09

**Authors:** Lou Bin, David McGiffin, Thuy Nguyen, Lv Wang, Yao Sun, Lumei Ye, Meiling Han, Chengju Sheng, Tzong-Hsien Lee, Marie-Isabel Aguilar, Anton Y. Peleg, Yue Qu

**Affiliations:** a Department of Laboratory Medicine, The First Affiliated Hospital, Zhejiang University School of Medicine, Hangzhou, Zhejiang Province, 310003, China; b Infection Program, Monash Biomedicine Discovery Institute, Department of Microbiology, Monash University, Clayton, Victoria, 3800, Australia; c Department of Cardiothoracic Surgery, The Alfred and Monash University, Melbourne, Victoria, 3004, Australia; d Critical Care Research Group, The Prince Charles Hospital, Brisbane, Queensland, Australia; e Department of Clinical Laboratory, The First Affiliated Hospital of Wenzhou Medical University, Wenzhou Medical University-Monash Biomedicine Discovery Institute Alliance in Clinical and Experimental Biomedicine, Wenzhou, Zhejiang Province, 325000, China; f Department of Materials Science and Engineering, Monash University, Melbourne, Victoria, 3800, Australia; g Department of Biochemistry and Molecular Biology, Monash University, Melbourne, Victoria, 3800, Australia; h Department of Infectious Diseases, The Alfred Hospital and School of Translational Medicine, Monash University, Melbourne, Victoria, 3004, Australia; i Centre to Impact AMR, Monash University, Melbourne, Victoria, 3800, Australia

**Keywords:** Staphylococcal biofilms, Antimicrobial resistance, Disk diffusion, Extracellular polymeric substance (EPS) matrix, And penetration

## Abstract

**Objectives:**

Limited antimicrobial penetration is an important mechanism underlying antimicrobial resistance of biofilms and has often been incorrectly quantitated. We adopted a rationalized antibiotic agar-diffusion model to accurately interpret experimental results of a widely-accepted biofilm penetration assay, and to determine drug-related physiochemical properties impacting antimicrobial biofilm penetration.

**Methods:**

Staphylococcal reference strains and eight conventional antibiotics were selected for this study. A well-established biofilm penetration assay based on disk diffusion and colony biofilms was used. Sizes of the zone of inhibition (ZOI) were converted to concentrations of antibiotics, using linear regressions of squared radii of the ZOI on the natural logarithm of antibiotic concentrations. Biofilm penetration ratios were calculated by comparing concentrations of antibiotics reaching the agar surface with or without biofilm barriers. Multiple regression analysis was performed to assess the impact of antibiotic physicochemical properties, such as surface charge, on their biofilm penetration.

**Results:**

Ciprofloxacin and oxacillin showed great capacities in penetrating staphylococcal biofilms. Rifampicin penetrated biofilms at low rates of ∼20 %. Aminoglycosides showed strain- and agent-specific penetration ratios, with tobramycin showing the least penetration (17.8 % for *Staphylococcus aureus* and 35.6 % for *Staphylococcus epidermidis*) and kanamycin presenting good penetration (∼82.3 %) against *S. aureus* biofilms. Surface charges of antibiotics at neutral and acidic conditions were important for their biofilm penetration.

**Conclusions:**

Accurate quantitation of antibiotic biofilm penetration can be achieved using the transformed linear regression between the ZOI and antibiotic concentrations. Mathematical evidence was provided to support the importance of surface charge of antimicrobials on their biofilm penetration.

## 1 Introduction

Microbial biofilms are the root cause of medical device-associated infections and often fail to respond to systemic antimicrobial therapies [1]. *Staphylococcus* species are predominant etiological agents of many device-related infections, particularly those related to orthopedic or cardiovascular devices [2,3]. The presence of staphylococcal biofilms has been implicated in many cases of treatment failure of medical device-related infections; these biofilms remained highly resistant to conventional antibiotics [2,4,5].

Vancomycin is still recommended and widely used for medical device-associated infections caused by methicillin-resistant *Staphylococcus* species [[6], [7], [8]], though numerous *in vitro* and *ex vivo* studies have reported minimal activity of vancomycin against staphylococcal biofilms [4,9]. Fluoroquinolone and rifampicin are antibiotics reserved for difficult-to-treat infections associated with staphylococcal biofilms [5], with uncertain efficacy and a significant incidence of side-effects [5,10]. Although resistance to aminoglycosides is a significant clinical issue in *Staphylococcus* species, treatment successes have been reported when gentamicin and/or tobramycin were used locally for prosthetic joint infections or vascular graft infections in which biofilm formation has been well documented [11,12].

A number of widely-accepted mechanisms underlie biofilm resistance to antibiotics, among them are the physiological and metabolic heterogenicity of biofilm cells, inoculum effects related to high-density growth mode such as quorum-sensing response and adaptative stress responses, the presence of persister cells, possible polymicrobial interactions, horizontal gene transfer, the presence of extracellular polymeric substance (EPS) matrix and accumulation of hydrolase-type enzymes that impede the diffusion of antibiotics to deeply-embedded biofilm cells [[13], [14], [15], [16]]. Numerous studies have been carried out to investigate the capacity of first-line or newly developed antimicrobial agents to penetrate biofilm EPS matrix, using either the disk-diffusion-based biofilm penetration assay [17,18], confocal laser scanning microscopy (CLSM) in combination with fluorescently tagged antibiotics [[19], [20], [21], [22], [23], [24]], or less frequently, the fluorescent polarization immunoassay [25]. The widely referenced disk-diffusion-based biofilm penetration assay uses the size of the zone of inhibition (ZOI) as the surrogate for the amount of antibiotics diffusing through biofilms and reaching the surface of agar medium pre-seeded with testing microorganisms [26]. Some studies directly compared the sizes of the ZOI in the presence or absence of biofilms for antibiotic biofilm penetration ratios [17,18]. Others converted the sizes of the ZOI to antibiotic concentrations in a way that was only vaguely described in their publications [13,14,27]. It is possible that these studies have used non-linear regressions to plot experimental ZOI for antibiotic concentrations.

Bonev et al. (2008) previously proposed transformed linear regression models that accurately predicted the concentration of active antibiotics based on the size of the ZOI [28]. Our study aimed to incorporate the well-fitting linear regression models by Bonev et al. (2008) into the biofilm penetration assay developed by Singh et al. (2010, 2016) [18,29], to accurately quantitate antibiotic penetration of staphylococcal biofilms and further evaluate the importance of physiochemical properties of antibiotics on their biofilm penetration.

## 2 Materials and methods

### 2.1 Microorganisms and antibiotics

Two biofilm-producing laboratory reference strains *Staphylococcus aureus* ATCC 25923 and *Staphylococcus epidermidis* RP62A and eight conventional antibiotics were selected for this study -oxacillin, ciprofloxacin, rifampicin, and five aminoglycosides, gentamicin, kanamycin, tobramycin, streptomycin, and amikacin (Sigma, USA). Vancomycin was not included due to its poor ability to penetrate through Muller-Hinton agar (MHA).

### 2.2 Establishing linear regressions between antibiotic concentrations and size of the ZOI

Liner regressions were established for each antibiotic and microorganism combination, using the radius or squared radius of the ZOI (r^2^) and the natural logarithm of antibiotic concentrations [ln (C)], following the principle published by Bonev et al. [28] (2008). The quadratic model [r^2^/ln (C)] and the simple liner model [r (radius)/ln (C)] were compared for the better linear fit when two staphylococcal strains were exposed to eight antibiotics respectively. The better fitted regression model was opted for to convert the size of the ZOI to concentrations of active antibiotics reaching the agar surface.

### 2.3 Antibiotic biofilm penetration assay

**Fig. 1:**
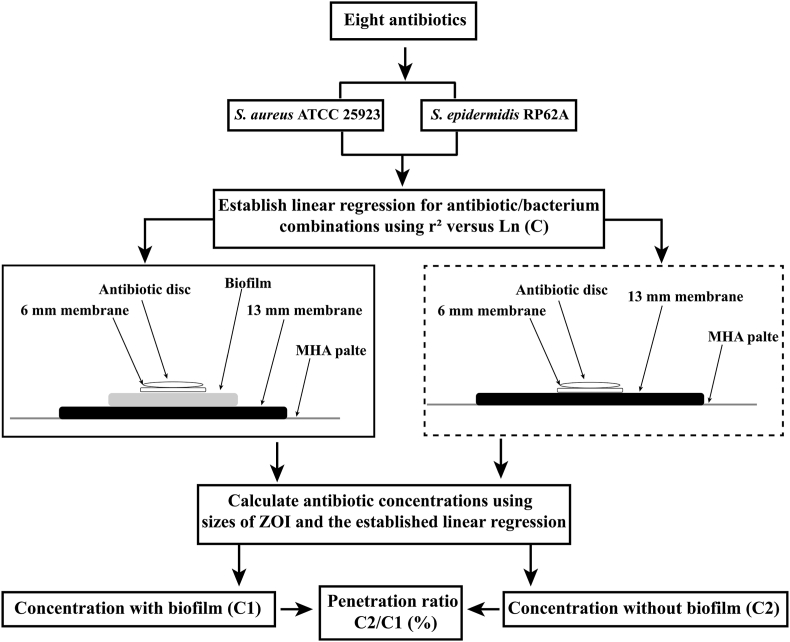
Schematic diagram of the core experimental design.

Diffusion of antibiotics through staphylococcal biofilms was quantitatively assessed using a biofilm penetration assay published by Singh et al. (2010), which combined colony biofilms with the disk diffusion assay [18,29]. To grow colony biofilms, 10 μL of bacterial suspensions prepared in tryptic soy broth were used to seed polycarbonate membranes (diameter, 13 mm; pore size, 0.4 μm) placed on tryptic soy agar plates, followed by incubation at 37 °C for 48 h. Colony biofilms formed by *S. aureus* ATCC 25923 and *S. epidermidis* RP62A have been qualitatively and/or quantitatively characterized in our previous studies [30,31] and those by others [17,29,32]. Antibiotics loaded in overlying filter paper disks diffused through staphylococcal biofilms, reaching a MHA surface pre-seeded with a lawn culture of staphylococcal cells and forming a ZOI after overnight growth. Sizes of the ZOI were further converted into concentrations of the antibiotics, using the pre-established quadratic model of liner regression. Control assemblies comprising antibiotic disks and sterile membranes without biofilms were tested in parallel on the same agar plate. The antibiotic biofilm penetration ratio was calculated as the concentration of antibiotics passing colony biofilms and reaching the agar surface divided by that passed the no-biofilm control assembly. A schematic diagram of the core experimental design was shown in Fig. 1.

### 2.4 Determining antibiotic biofilm penetration using UPLC-MS/MS

The colony biofilm sandwich assay published by Anderl et al. (2000) [14] was adopted to assess antibiotic biofilm penetration using Liquid Chromatography-Tandem Mass Spectrometry (LC-MS/MS). 48-h-old colony biofilms were grown on 25-mm-diameter microporous polycarbonate membranes and placed on MHA plates containing 100 μg/mL of ciprofloxacin or 10 μg/mL of gentamicin. A 13-mm-diameter microporous polycarbonate membrane was placed on top of the biofilm, with a 6-mm-diameter moistened concentration disk sitting on top of the membrane. The entire set-up was incubated at 37 °C for 16 h to allow antibiotics to diffuse from the MHA to the concentration disks. Antibiotics in the disks were further released into 500 μL of PBS. The concentrations of ciprofloxacin and gentamicin in PBS were analyzed using ultraperformance LC-MS/MS (UPLC-MS/MS, Shimadzu) in positive electrospray ionisation mode. Detection was performed in multiple reaction monitoring (MRM) mode using the following transitions: *m/z* 332.1/288.1 for ciprofloxacin and *m/z* 478.3/322.2 for gentamicin. The limit of quantification was 0.05 μg/mL for each analyte.

### 2.5 Zeta-potential measurement

Surface charges of biofilm EPS matrix and antibiotics were assessed by measuring zeta-potentials of these materials prepared in phosphate buffered saline (PBS) at pH = 5.5 and pH = 7.4, representing an acidic inner-biofilm microenvironment and a neutral biofilm-surrounding microenvironment such as the bloodstream. To test the surface charge of the biofilm EPS, colony biofilms were grown on 25 mm membrane filters placed on Tryptic Soy Agar (TSA) plates for 24 h and transferred into a sterile tube with 2 mL of PBS using a sterile cotton swab. The biofilm EPS matrix was isolated using a previously published method [33]. Zeta-potentials of biofilm matrix and antibiotic solutions were measured using ZEN3600 (Malvern Panalytical, UK). The measurement was carried out for three independent samples.

### 2.6 Statistical analysis

All experiments except zeta-potential measurement were carried out in three biological repeats in technical triplicate. Two-sample *t*-test was carried out to compare the penetration ratios determined using UPLC-MS/MS and the optomized ZOI method respectively. Linear regressions were established using the software Excel (Microsoft, USA). Multiple regressions for antibiotic biofilm penetration ratios and physiochemical properties were analyzed. Minitab Statistical Software 16 for Windows (Minitab Ltd., Coventry, UK) was used for statistical analysis, with a significance level of 0.05.

## 3 Results

### 3.1 A strong linear fit between the squared radii of the ZOI and ln concentrations were found for all tested antibiotics and microorganisms

**Fig. 2:**
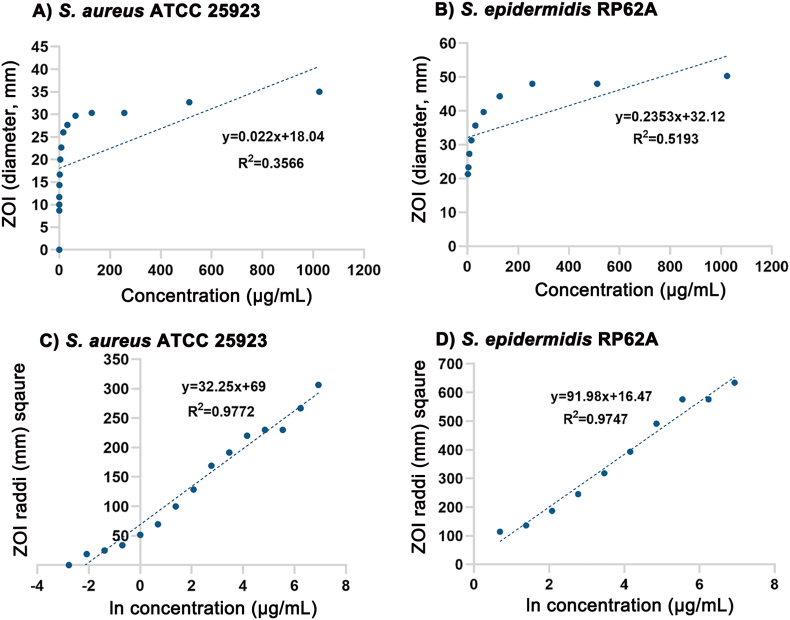
Comparisons of linear regression fits of a simple model utilizing the diameter of the zone of inhibition (ZOI) and rifampicin concentration (A & B) and a transformed model using squared radii and the natural log (ln) of concentrations (C & D).

We first evaluated the linear regression between the concentration of antibiotics added to filter paper disks in the disk diffusion assay and the measured diameter of the ZOI, using rifampicin as the representative antibiotic. Poor linear regressions were observed when the two parameters, antibiotic concentrations and ZOI diameters, were plotted directly against each other (Fig. 2A and B), with a coefficient of determination (R2) of 0.3566 for *S. aureus* ATCC 25923 and 0.5193 for *S. epidermidis* RP62A respectively. After transformation of the two parameters, a strong linear regression was seen between the natural logarithm of concentrations [ln (C)] and the ZOI radii square (r^2^) (Fig. 2C and D).

**Fig. 3:**
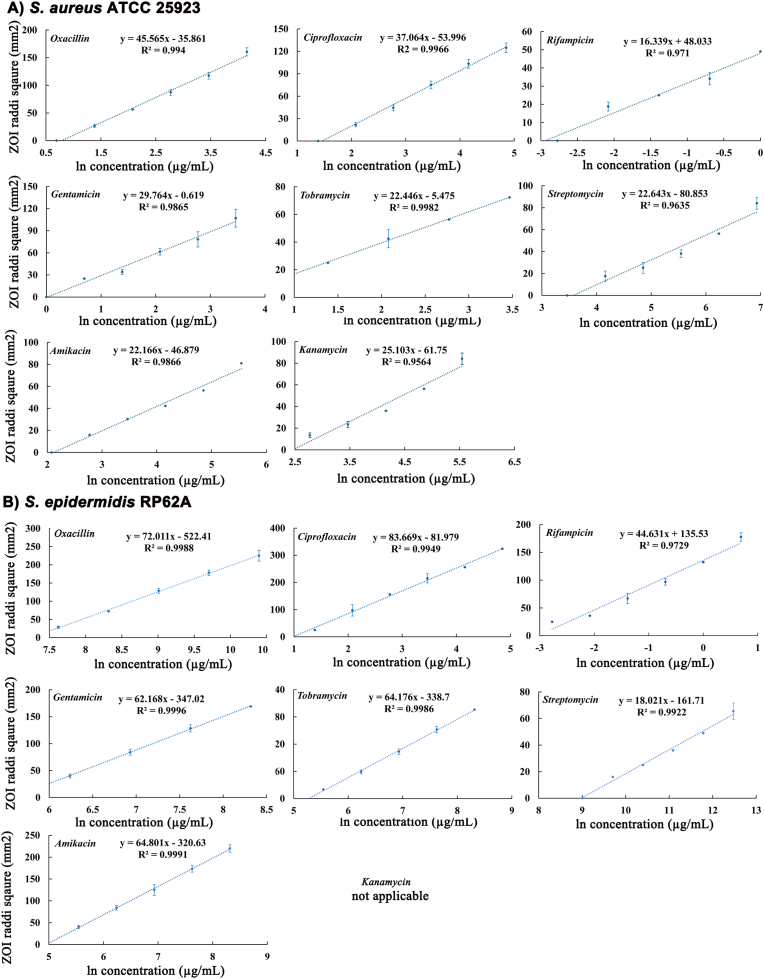
Linear regressions of squared radii of the zone of inhibition (ZOI) on the natural log (ln) of concentrations established for 15 different microorganism and antibiotic combinations. The experiment was carried out in three biological repeats in technical triplicate. The error bars represent standard errors of the mean. No linear regression was established for kanamycin against *S. epidermidis* RP62A as the microorganism is highly resistant to the antibiotic and no ZOI was formed.

With ln (C) as the explanatory variable, Bonev et al. (2008) used the ZOI radii (r, simple linear model) and squared ZOI radii (r^2^, quadratic model) respectively as the dependent variable and reported slightly different and antibiotic-dependent linear fits [28]. With experimental data obtained in this study, we established 15 linear regressions for different antibiotic/microorganism combinations for each model and further compared overall linear fits or R^2^ for these two models. Our results showed that 12 out of 15 antibiotic/microorganism combinations had slightly better linear fits or higher R^2^ for the quadratic model using the ZOI radii square (Table 1). Thus, the quadratic model was used to calculate antibiotic concentrations from the ZOI radii for all tested antibiotics and microorganisms (Fig. 3).

**Table 1:** Linear fits of different regression models for antibiotic/microorganism combinations.

Bacterium & regression models	Liner fits or the coefficient of determination (R^2^)							
	Oxa	Cip	Rif	Gen	Kan	Tob	Amk	Stp
***S. aureus* ATCC 25923**								
**r^2^/ln (C) model**	0.994	0.9966	0.971	0.9865	0.9557	0.9982	0.9866	0.9635
**r/ln (C) model**	0.938	0.9325	0.842	0.9104	0.9929	0.9888	0.9174	0.9283

***S. epidermidis* RP62A**								
**r^2^/ln (C) model**	0.9988	0.9949	0.9729	0.9996	NA^a^	0.9986	0.9991	0.9922
**r/ln (C) model**	0.9822	0.9497	0.9948	0.9863	NA	0.9859	0.9903	0.9997

a Not available as no zone of inhibition was observed. Oxa, oxacillin; Cip, ciprofloxacin; Rif, rifampicin; Gen, gentamicin; Kan, kanamycin; Tob, tobramycin; Amk, amikacin; Stp, streptomycin. r, radius; C, concentration.

### 3.2 Accurate quantitation of staphylococcal biofilm penetration of eight conventional antibiotics

We assessed biofilm penetration capacities of eight conventional antibiotics using the disk diffusion assay in combination with colony biofilms. The sizes of the ZOI were converted to antibiotic concentrations using the linear regressions previously formulated. The biofilm penetration ratio of antibiotics was calculated using the formula listed below and presented in Table 2.

$$\text{Biofilm}\;\text{penetration}\;\text{ratio}=\frac{\text{Concentration}\;\text{of}\;\text{antibiotics}\;\text{passing}\;\text{biofilms}\;\text{and}\;\text{reaching}\;\text{the}\;\text{agar}\;\text{surface}\;}{\text{Concentration}\;\text{of}\;\text{antibiotics}\;\text{reaching}\;\text{the}\;\text{agar}\;\text{surface}\;\text{without}\;\text{biofilm}\;\text{barriers}}$$

**Table 2:** Quantitative assessment of antibiotic penetration through staphylococcal biofilms.

Bacterium	*S. aureus* ATCC 25923				*S. epidermidis* RP62A			
Antibiotic	Amount of antibiotic added to filter paper disk (μg in 20 μL PBS)	Concentration of antibiotics reaching agar surface (μg/mL)		Biofilm penetration ratio	Amount of antibiotic added to filter paper disk (μg in 20 μL PBS)	Concentration of antibiotics reaching agar surface (μg/mL)		Biofilm penetration ratio
		No biofilm control (C1)	With biofilm barrier (C2)	C2/C1 (%)		No biofilm barrier (C1)	With biofilm barrier (C2)	C2/C1 (%)
**Oxacillin**	1.28	26.9 ± 3.8	26.9 ± 3.8	100.0 ± 14.1	327.68	4953.6 ± 0.0	3323.4 ± 202.7	67.1 ± 4.1
**Ciprofloxacin**	0.64	24.1 ± 0.0	21.1 ± 2.6	87.4 ± 10.9	0.64	27.7 ± 0.0	19.1 ± 1.6	69.0 ± 5.9
**Rifampicin**	0.04	4.9 ± 2.5	0.8 ± 0.2	18.7 ± 4.7	0.04	1.1 ± 0.2	0.3 ± 0.0	24.9 ± 2.7
**Gentamicin**	0.64	8.8 ± 0.0	4.2 ± 0.0	48.2 ± 0.0	40.96	1564.9 ± 0.0	849.2 ± 0	54.3 ± 0.0
**Kanamycin**	10.24	149.8 ± 0.0	123.3 ± 23.0	82.3 ± 15.3	10.24	NA	NA	NA
**Tobramycin**	1.28	47.1 ± 0.0	8.4 ± 0.0	17.8 ± 0.0	40.96	2729.3 ± 0.0	971.3 ± 394.0	35.6 ± 14.4
**Amikacin**	5.12	215.8 ± 0.0	104.9 ± 0.0	48.6 ± 0.0	20.48	1570.5 ± 0.0	597.9 ± 53.2	38.1 ± 3.4
**Streptomycin**	40.96	1913.2 ± 0.0	911.9 ± 338.3	47.7 ± 17.7	20.48	NA	NA	NA

NA, not available due to the strain being highly resistant to the antibiotic or there was no ZOI. PBS, phosphate buffer saline.

Among the eight antibiotics, oxacillin and ciprofloxacin showed excellent capacities to penetrate biofilms formed by both *S. aureus* ATCC 25923 and *S. epidermidis* RP62A, with penetration ratios ranging from 67.1 ± 4.1 % to 100 ± 14.1 %. Variable penetration was observed for aminoglycosides, with average ratios ranging from 17.8 % to 82.3 %. Tobramycin had the lowest penetration rates of 17.8 ± 0.0 % for *S. aureus* biofilms and 35.6 ± 14.4 % for *S. epidermidis* biofilms. Kanamycin had the greatest penetration (82.3 ± 15.3 %) through biofilms formed by *S. aureus* ATCC 25923. Gentamicin, amikacin, and streptomycin all demonstrated ∼50 % penetration through biofilms formed by *S. aureus* ATCC 25923. Biofilm penetration of kanamycin and streptomycin were not assessed; *S. epidermidis* RP62A is resistant to both antibiotics and a ZOI was not observed on MHA plates. Limited biofilm penetration was observed for rifampicin, with less than 25 % of active drugs passing staphylococcal biofilms.

### 3.3 Validating ciprofloxacin and gentamicin penetration through S. aureus biofilms using UPLC-MS/MS

Three antibiotics classified as high, moderate, and low penetration through *S. aureus* ATCC 25923 biofilms by the optimized ZOI method, including ciprofloxacin, gentamicin and rifampicin were initially selected for UPLC–MS/MS analysis. Our preliminary assessment of rifampicin revealed two distinct peaks. Rifampicin has been reported to undergo oxidation when dissolved in methanol and form a quinone derivative; this oxidative degradation affects its stability and quantification when analyzed by LC-MS/MS [34]. It was thus decided to only include ciprofloxacin and gentamicin for the UPLC-MS/MS analysis. Using UPLC-MS/MS, ciprofloxacin and gentamicin were found to penetrate *S. aureus* ATCC 25923 colony biofilms at ratios of 88.0 ± 4.4 % and 53.3 ± 6.6 %, respectively, consistent with the values obtained using the optimized ZOI method (Table 3).

**Table 3:** Antibiotic penetration through *S. aureus* ATCC 25923 biofilms determined by UPLC-MS/MS and compared to that obtained using the optimized ZOI method.

Antibiotic	UPLC-MS/MS			Optimized ZOI method	*P-*value
	No biofilm control (μg/mL)	With biofilm barrier (μg/mL)	Penetration ratios (%)	Penetration ratios (%)	
Ciprofloxacin	6.51 ± 0.63	5.72 ± 0.36	88.0 ± 4.4	87.4 ± 10.9	0.694
Gentamicin	0.52 ± 0.05	0.28 ± 0.06	53.3 ± 6.6	48.2 ± 0.0	NA

UPLC-MS/MS, Ultraperformance Liquid Chromatography-Tandem Mass Spectrometry; ZOI, zone of inhibition. NA, not applicable due to all values of the concentration determined using the optimized ZOI method were identical; this concentration still falls in the concentration range determined with UPLC-MS/MS.

### 3.4 Antibiotic surface charges impact their staphylococcal biofilm penetration

Taking advantage of accurate quantitation of antibiotic biofilm penetration, we further analyzed the impacts of different physiochemical properties of antibiotics on their biofilm penetration ratios, including surface charge as zeta-potentials, molecular size, and solubility in water. The EPS matrix of colony biofilms formed by *S. aureus* ATCC 25923 had zeta potentials of −10.8 ± 1.2 mV and −8.44 ± 0.68 mV when prepared as suspensions in PBS with a neutral pH (7.2) and a weakly acidic pH (5.5) respectively, and that for *S. epidermidis* RP62A biofilms were −11.0 ± 1.2 mV (pH = 7.2) and −10.00 ± 0.03 mV (pH = 5.5), indicating the negatively charged nature of staphylococcal biofilm EPS. We also assessed zeta-potentials of antibiotics at the concentrations that were used for biofilm penetration assays. These antibiotic solutions were prepared in PBS adjusted to pHs of 7.2 and 5.5 respectively, representing the surface and inner-biofilm microenvironments. A multiple regression analysis of biofilm penetration ratios and physiochemical properties of antibiotics was established as below, with an overall F-statistic in the regression output close to a significant level (*p* = 0.069) and an adjusted R^2^ of 40.2 %.

Biofilm penetration ratio = 3.64ζ_7.2_–13.2ζ_5.5_–0.027 M.W. + 0.167Ksp_(H2O)_ + 84.2

Here 84.2 is the constant, ζ_7.2_ and ζ_5.5_ are zeta potentials at pH = 7.2 and pH = 5.5. M.W. refers to molecular sizes of the drug, and Ksp_(H2O)_ is the water solubility.

Among the three physiochemical properties, zeta potentials of antibiotics at neutral (ζ_7.2_) and acidic conditions (ζ_5.5_) appeared to significantly correlate with the drug penetration of biofilms, supported by *p* values of 0.052 and 0.016 respectively (Table 4). No significant correlation was found between the molecular size or water solubility of the antibiotics and their biofilm penetration.

**Table 4:** Impacts of antibiotic-related physical-chemical properties on antibiotic biofilm penetrations.

Antibiotics	Concentration in the drug disks	Biofilm penetration ratio (%)		Zeta-potential (surface charge)		Molecular size (g/mol)	Water solubility (mg/mL)
		*S. aureus* ATCC 25923	*S. epidermidis* RP62A	pH = 7.2	pH = 5.5		
**Oxacillin**	32 μg/mL;	100.0 ± 0.0	NA	−5.05 ± 1.47	−1.21 ± 0.84	423.42	10
	32,768 μg/mL	NA	67.1 ± 4.1	−1.61 ± 0.16	0.08 ± 0.06		
**Ciprofloxacin**	32 μg/mL	87.4 ± 10.9	69.0 ± 5.9	−0.03 ± 0.06	0.17 ± 0.09	331.34	3
**Rifampicin**	2 μg/mL	18.7 ± 4.7	24.9 ± 2.7	−10.02 ± 1.24	0.06 ± 0.06	823.00	25
**Gentamicin**	32 μg/mL	48.2 ± 0.0	NA	−9.69 ± 2.10	0.09 ± 0.04	463.00	10
	4096 μg/mL	NA	54.3 ± 0.0	−0.99 ± 0.21	2.38 ± 0.57		
**Kanamycin**	512 μg/mL	82.3 ± 15.3	NA	−5.93 ± 0.94	0.13 ± 0.03	582.60	50
**Tobramycin**	64 μg/mL	17.8 ± 0.0	NA	1.74 ± 0.06	2.83 ± 0.69	467.51	50
	4096 μg/mL	NA	35.6 ± 14.4	1.76 ± 0.54	4.22 ± 1.02		
**Amikacin**	256 μg/mL	48.6 ± 0.0	NA	−10.2 ± 1.25	−1.88 ± 0.56	781.80	50
	4096 μg/mL	NA	38.1 ± 3.4	−7.55 ± 1.01	−1.43 ± 0.70		
**Streptomycin**	2048 μg/mL	49.3 ± 0.0	NA	0.16 ± 0.07	0.86 ± 0.24	728.69	100

NA, not available.

## 4 Discussion

Limited penetration of antibiotics through biofilm matrix is a well-established mechanism that underlies antimicrobial resistance of microbial biofilms [35]. Its importance in biofilm resistance to conventional or newly developed antimicrobials, however, were often falsely estimated, partially due to the lack of a consistent method that allows accurate and quantitative assessment of antimicrobial penetration of biofilms [13,18].

Numerous studies have intended to quantitatively or semi-quantitatively assess antibiotic biofilm penetration, mostly using the disc-diffusion-based biofilm penetration assay. It is possible that a simple linear regression directly comparing the ZOI diameter and the minimum inhibitory concentration (MIC) breakpoints for antibiotic susceptibility [36,37] have been mistakenly used to convert the sizes of the ZOI into antibiotic concentrations for the calculation of biofilm penetration ratios. We combined the widely used disk-diffusion-based biofilm penetration assay [29] and a transformed linear regression using the natural logarithm of antibiotic concentration and the squared radii of the ZOI, and provided accurate quantitation of antibiotic biofilm penetration. Ciprofloxacin is a preferred antibiotic for medical device-related staphylococcal infections due to its promising activities against biofilms [5]. This antibiotic is neutrally charged at physiological pH and has an excellent capacity in penetrating staphylococcal biofilms, as found in the current study and by others [18,29]. Rifampicin is controversial as a single or combinational therapy for staphylococcal biofilm infections [38] and it has been recommended as an adjunct agent for medical device-associated staphylococcal infections [5,38,39]. Despite the popular clinical belief that rifampicin can readily penetrate staphylococcal biofilms and kill biofilm cells, a relatively low capacity for biofilm penetration was found for this drug in our study. Zheng and Steward used the ZOI to evaluate rifampicin penetration through *S. epidermidis* RP62A colony biofilms and reported a penetration ratio greater than 90 % after 12 h [27]. These authors might have converted the ZOI into rifampicin concentrations using a poor linear regression similar to what we showed in Fig. 2B and consequentially obtained a falsely high ratio [27]. The high ratio of maximum serum concentration to the minimum inhibitory concentration (MIC) (Cmax/MIC) [40] may compensate for limited penetration of rifampicin through staphylococcal biofilms and support it's *in vivo* or clinical efficacy. Interestingly, a recently published multicenter Randomized Controlled Trial (RCT) found no additional effect of rifampicin in antibiotic combinations for treating periprosthetic joint infection [38]. Aminoglycosides at high concentrations have been used through local application to treat prosthetic join infections or ventricular assist device driveline infections, often in combination with vancomycin and delivered by antibiotic beads [11,41]. Most aminoglycosides showed moderate biofilm penetration in our study. Tobramycin and gentamicin are two aminoglycosides that have been used in STIMULAN Rapid Cure beads for bone and joint infections [11]. Although other aminoglycosides such as kanamycin and amikacin were able to penetrate through staphylococcal biofilms without significant impediment, as found in this study and by others [18,29], the widely distributed antimicrobial resistance of these antibiotics in staphylococcal isolates may limit their clinical application for biofilm-related infections. Vancomycin is another recommended antibiotic for use with STIMULAN Rapid Cure beads, particularly in the treatment of biofilm-associated infections. We were unable to accurately assess vancomycin biofilm penetration using the method described in this study due to the limited diffusion of this antibiotic through MHA and failure to form a clear ZOI. Many other studies using fluorescently tagged vancomycin and confocal laser scanning microscopy have reported very poor penetration of vancomycin through staphylococcal biofilms [18,19,42],

Antibiotic biofilm penetration is a complex interplay of the properties of antibiotics and the characteristics of biofilms [14,35,43]. Although being antibiotic- and microorganism-specific [18,27], it is generally believed that the penetration ratio is relevant to the size, charge, and hydrophobicity of antibiotics, with smaller, neutral, and hydrophobic antibiotics diffusing more easily through the negatively-charged biofilm EPS matrix. We studied the importance of three key physicochemical properties of antibiotics on their biofilm penetration, including surface charge, molecular size and solubility. Our multiple regression analyses suggests that the surface charge of antibiotics at neutral and weakly acidic pH levels significantly affects their ability to penetrate biofilms; the former represents the microenvironment in which drugs initially bind to the biofilm surface and the latter is for an inner-biofilm microenvironment. Under an weakly acidic inner-biofilm condition, anionic interactions between positively-charged antibiotics and negatively-charged biofilm EPS was proposed as a major reason that limited biofilm penetration [44]. It is thus not surprising that the neutral antibiotics ciprofloxacin and oxacillin were able to freely penetrate biofilms, while tobramycin, an aminoglycoside with positively charged amino-groups, were bound electrostatically to the negatively charged staphylococcal biofilm EPS and showed low biofilm penetration [45].

Biofilm infection sources are often characterized by poor antibiotic penetration and low bacterial metabolic activities [46]. Two prerequisites must be met to allow antibiotics to successfully eradicate microbial biofilms: Adequate antibiotics reaching deeply embedded biofilm cells and the capability of antibiotics to kill metabolically inactive cells. Inadequate penetration of antibiotics into biofilms often leads to the formation of antibiotic concentration gradients within the biofilm. This not only fails to kill biofilm-associated cells but poses a risk of enhancing biofilm formation due to the presence of antibiotics at sub-inhibitory concentrations [47,48]. Although oxacillin showed an excellent capacity to move through biofilms, the bactericidal activity of β-lactams strongly relies on active bacterial metabolic status [49]. This may explain the lack of efficacy of oxacillin in killing metabolically inactive staphylococcal biofilm cells. In addition to the biofilm penetration, the metabolism-dependence of antibiotics needs to be considered when developing an arsenal of antibiotics for biofilm-associated infections.

Caution must be exercised when setting up a biofilm penetration assay. Specific transformed linear regression models must be pre-established across different microorganism-antibiotic combinations to allow accurate conversion of the sizes of the ZOI to antibiotic concentrations. The disk-diffusion based biofilm penetration assay should not be used if the microorganism-antibiotic combination fails to form a clear ZOI on MHA plates, i. e when the microorganism is highly resistant to the antibiotic or when the antibiotic has a large molecular weight and a limited diffusion through MHA. Accurate measurement of the ZOI is also critical for the quantitation of antibiotic biofilm penetration. The size of the ZOI measured has an exponential impact on the corresponding antibiotic concentration [Antibiotic concentration = e^B^ * ^(r2 or r)−A^, where r is the radius and A and B are constants related to the antibiotic and microorganism]. A random error of 1 mm in the ZOI measurement may lead to a significant change in antibiotic concentrations. In the disk-diffusion assay, the initial inoculum of the microorganism and the thickness of the agar medium both significantly affect the size of the ZOI. This can be addressed if the experimental and control assemblies were set up on the same agar plate and a caliper is used for the measurement.

This study has limitations. Only two laboratory reference strains and no clinical isolates were included in this study. The conclusion drawn from this study was based on static colony biofilms, while other biofilm models, including those involving a dynamic set-up [10,[20], [21], [22],^25^], may produce different results and need to be further investigated. Although eight conventional antibiotics were studied, five of them were aminoglycosides. Antibiotics of other classes, such as daptomycin and minocycline, should be assessed in the future.

## CRediT authorship contribution statement

**Lou Bin:** Writing – review & editing, Validation, Methodology, Investigation, Formal analysis, Data curation, Conceptualization. **David McGiffin:** Writing – review & editing, Validation, Supervision, Investigation, Funding acquisition, Formal analysis, Data curation. **Thuy Nguyen:** Writing – review & editing, Formal analysis, Data curation. **Lv Wang:** Data curation, Methodology, Writing – review & editing. **Yao Sun:** Writing – review & editing, Formal analysis, Data curation. **Lumei Ye:** Writing – review & editing, Formal analysis, Data curation. **Meiling Han:** Data curation, Writing – review & editing. **Chengju Sheng:** Writing – review & editing, Formal analysis, Data curation. **Tzong-Hsien Lee:** Writing – review & editing, Formal analysis, Data curation. **Marie-Isabel Aguilar:** Writing – review & editing, Supervision, Investigation, Formal analysis. **Anton Y. Peleg:** Writing – review & editing, Supervision, Funding acquisition, Formal analysis, Conceptualization. **Yue Qu:** Writing – review & editing, Writing – original draft, Validation, Supervision, Resources, Project administration, Methodology, Investigation, Funding acquisition, Formal analysis, Data curation, Conceptualization.

## Disclosure statement

No authors have no conflicts of interest to disclose. None of the authors have a financial relationship with a commercial entity that has an interest in the subject of the presented manuscript or other conflicts of interest to disclose. The authors acknowledge the financial assistance provided by the Department of Infectious Diseases, and Department of Microbiology, Monash University, Australia. The funding organizations listed above had no role in the collection of data, its analysis and interpretation, and in the right to approve or disapprove publication of the finished manuscript.

## Funding

This work was supported by Leading Geese Research and Development Plan of Zhejiang Province (No. 2024C03218), MRFF Artificial Heart Frontier Program and the Monash Institute of Medical Engineering (to Y. Q., D.M. and A.P.), and National Key R&D Program of China (2024YFC2707400).

## Declaration of competing interest

The authors declare that they have no known competing financial interests or personal relationships that could have appeared to influence the work reported in this paper.

## Data Availability

Data will be made available on request.
